# Correspondence

**Published:** 1907-10

**Authors:** E. R. Anthony

**Affiliations:** Secretary of Board


### CORRESPONDENCE.

Mr. Editor.—Having been a member of the Georgia Board of Medical Examiners for several years, I feel that I know something of the defects of the law under which the board has been working.

Since the enactment of the law in 1894 creating this board, there has been but one amendment to it—the amendment "allowing the Board to reciprocate in the matter of medical license with other States having the same standard of examinations and equal requirements."

There is an urgent necessity, I think, for other amendments, more important than the one just named—amendments which I hope will meet with the approval of the Committee on Legislation of the Medical Association of Georgia, and I respectfully submit them to that committee. The first amendment, I suggest, is to change the manner of appointing the members of the Board. As the law now stands the Governor appoints. I suggest, as an amendment that when a vacancy occurs by expiration of the term for which the member was appointed, that the Governor appoint a successor from three names to be selected and furnished him, by the Medical Association, of Georgia.

In making this suggestion I mean no reflection whatever, on the Governor's appointments hereto-fore made. I have had the honor of serving on the Board with every appointee since this law was enacted, and do not believe that more excellent appointments could have been made. All the members of the Board have been appointed from the very best material of our honored profession in this State, and I am satisfied that the appointments made have been satisfactory to our Association.

In support of this suggestion, I will say, that I do not believe that any one has the moral right, nor should he have the ambition, to serve on this Board, who can not get the endorsement of the Medical Association of Georgia, consisting as it does of 1500 to 2000 of the best men in the profession.

While it is true, as Georgia's greatest jurist, the late Hiram Warner, said, "that very few office holders die" and none resign", still vacancies may occur through resignation or death. In such cases the Governor might still appoint the successor for the unexpired term, free from the suggestions of the Medical Association of Ga.

Secondly, I suggest that the law be amended in the matter of granting temporary licenses. As it now is, a young man wanting temporary license is compelled to tour the State and visit three members of the Board at their respective homes, for oral examination,—entailing upon him a great loss of time, and large expense, which he is usually unable to bear. I suggest that the law be amended so that temporary license can be granted after an examination, either by the President of the Board, or by the Secretary, who is really the executive officer of the Board.

Thirdly, under the present law, the Board is compelled to have only two meetings—one immediately after the closing exercises of the Atlanta and Augusta Schools—the other second Tuesday in October. I suggest an amendment providing a third meeting of the Board about the first of July, for the purpose of accommodating those who graduate out of the State—the Tulane, Jefferson and Baltimore, and other colleges having their closing exercises a month or two later than the Atlanta colleges. This is but justice to the out-of-the-State graduates.

Fourthly, I suggest as a further amendment, that power be given the Board to revoke a license for just cause shown. If one should fraudulently procure a license, or should act in such a way as to bring reproach upon the profession, his license should be revoked.

Fifthly, I suggest an amendment that requires a higher preliminary education in an applicant for medical license. No license should be issued unless the applicant passes an examination in all the branches in which the teachers of our high schools are examined: unless the applicant holds a diploma from a medical college which requires this preliminary examination: or a certificate or diploma from a high school or college, in which event this examination will not be required. Let the first day of the Board's meeting be devoted to such examinations, and the session of the board be extended from two days to three days session. I feel assured that every member of our Board would gladly endorse this suggestion for the good of our profession.

I am very truly,
E. R. ANTHONY,
Secretary of Board.

